# Assessment of oral hypofunction and its association with age among Korean community-dwelling older adults

**DOI:** 10.1186/s12903-024-04180-2

**Published:** 2024-04-10

**Authors:** Hye-Jin Park, Eun-Ha Jung, Soo-Min Kim, Seong-Chan Park, Min-Ji Jo, Yun-Seon Lee, Sung-Hoon Kim, Sun-Young Han

**Affiliations:** 1 Oral Science Laboratory, Department of Dental Hygiene, College of Software and Digital Healthcare Convergence, Yonsei University, Wonju, Republic of Korea; 2 Department of Dental Hygiene, College of Software and Digital Healthcare Convergence, Yonsei University, Wonju, Republic of Korea; 3 Department of Rehabilitation Medicine, Yonsei University Wonju College of Medicine, Wonju, 26426 Republic of Korea

**Keywords:** Older adults, Oral frailty, Oral hypofunction, Oral health, Oral rehabilitation

## Abstract

**Background:**

Due to the increasing proportion of older adults in Korea and growing interest in aging, the concepts of oral aging and oral hypofunction have recently been introduced. Thus, it is necessary to investigate the age-specific oral function levels of Korean older adults and develop expert intervention methods for healthy aging.

**Methods:**

Dysphagia, independence of daily living, and oral hypofunction were assessed in 206 older adults living in Wonju, Gangwon State, South Korea. Subjective dysphagia was assessed through self-report questionnaires using the Dysphagia Handicap Index (DHI), the Korean version of Eating Assessment Tool-10, and the Korean version of the Modified Barthel Index. In addition, the oral hypofunction assessment items included decreased chewing ability, occlusal pressure, tongue pressure, oral dryness, and oral cleanliness.

**Results:**

DHI increased significantly with age, with those in their 80 s reporting the most difficulty swallowing. Oral function in terms of chewing ability (maximum occlusal pressure and number of remaining teeth), maximum occlusal pressure, and maximum tongue pressure also declined with increasing age. While there was no significant difference in oral dryness by age, those in their 80 s had dry mouth according to the criteria of the oral moisture checking device.

**Conclusions:**

In an assessment of oral function in community-dwelling, independent Korean older adults, the number of items that were assessed as oral hypofunction increased with age. The findings can be used to standardize the oral hypofunction assessment item and develop age-based individualized intervention plans for the early management of oral health and individual oral myofunctional rehabilitation in Korean community-dwelling older adults.

## Background

The increased attention to health and advances in living standards has extended human lifespan, resulting in a growing proportion of the world’s older adult population [1]. In Korea, the number of older adults aged 65 years and over is reported to be 17.5% as of 2022, and it is expected to become a super-aged society when this number reaches 20.3% in 2025; thus, there has been a growing interest in aging in relation to the health and quality of life of older adults [2].

Frailty is a biophysical disorder that affects daily life and an important consideration in extending life expectancy. Oral frailty has recently been recognized as an important factor in frailty. Oral frailty is defined as oral symptoms characterized by poor oral hygiene, slurred speech, throat clearing during meals, food spillage, and an increased number of foods that cannot be chewed, contributing to systemic frailty through mechanisms of inflammation and poor nutrition [3, 4]. Prolonged oral frailty can particularly lead to oral dysfunction, which is an irreversible condition that can affect systemic physical dysfunction [5]. In Korea, where the population is continuously aging, it is necessary to understand oral frailty and oral hypofunction in older adults and make efforts to prevent and alleviate them.

Chewing ability decreases with aging due to various geriatric conditions, medications, and dry mouth. Moreover, the muscle strength required for swallowing decreases in relation to sarcopenia, leading to dysphagia (swallowing disorder). Other contributing factors to dysphagia in older adults include certain systemic diseases (stroke, Parkinson’s disease, etc.), decreased chewing ability, and dry mouth due to changes in salivary properties [6, 7]. A previous study found that the risk of malnutrition was 4.8 times higher among those with reduced chewing ability and dysphagia compared to those without [8].

Other countries with rapidly aging populations that have reported changes in the oral status of older adults due to oral frailty and oral hypofunction [9, 10] have shown interest in oral rehabilitation and cleanliness to ensure that older adults can lead a healthy life. However, research on the oral function of older adults in Korea are still scarce, and the Korean diagnostic criteria for oral frailty were only recently published by the Korean Academy of Geriatric Dentistry (KAGD) [11]. Furthermore, since aging and frailty may reflect the characteristics of the population, it is necessary to identify the level of oral function in Korean community-dwelling older adults and develop appropriate interventions.

Therefore, this study aimed to assess the age-specific levels of dysphagia, independence of daily living, and major oral hypofunction among community-dwelling older adults in Korea to provide a basis for developing an oral function rehabilitation program based on the characteristics and age of Korean older adults.

## Methods

### Participants

This study was conducted from January 15 to February 24, 2023 among older adults living in Wonju, Gangwon State. The sample size was calculated using the G*Power program 3.1.9.4 (Heinrich-Heine-Universitat Dusseldorf, Germany) with an effect size of 0.25, significance level of 0.05, and power of 90%. Consequently, at least 207 participants were required. Finally, the final sample size was determined to be 217 participants considering a 5% dropout rate. Participants were selected among older adults aged 60 years or older living in Wonju, Gangwon State. Prior to recruiting participants, the research team visited senior centers in Wonju City and spoke with the person in charge. During the study period, seniors who visited the senior center provided written consent and completed an oral hypofunction survey or test.

**Fig. 1:**
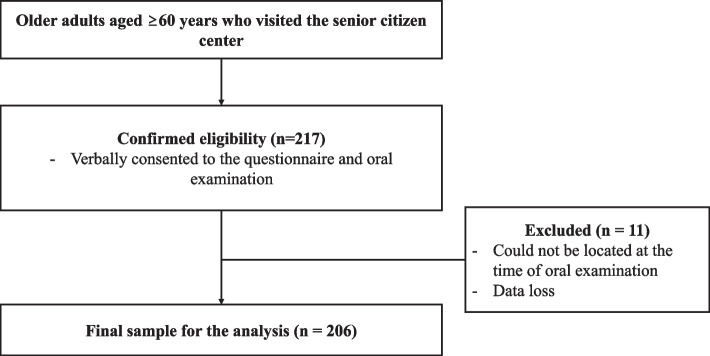
Flowchart of the selection of participants

The inclusion criteria were as follows: (1) aged 60 years or older; and (2) able to respond to the questionnaire and oral function test due to a precise level of consciousness or ability to judge their own cognitive ability. Subjects were excluded if they were unable to respond to the questionnaire and oral function test due to an unclear level of consciousness or inability to judge their own cognitive ability (Fig. 1).

### General participant characteristics

In order to characterize oral hypofunction, age, gender, presence of systemic diseases, and denture wear of the study participants were surveyed. The height and weight of the participants were also measured to determine their nutritional intake and calculate their body mass index (BMI, kg/m^2^). BMI was categorized into four levels: underweight (< 18.5), normal weight (18.5 ≤ BMI < 23), pre-obese (23 ≤ BMI < 25), and obese (25 ≤). The information about systemic diseases was obtained through open-ended questions: “Do you have any medical conditions that has been diagnosed by a specialist? If so, what condition is it?” Denture wearers were classified into those using upper and lower partial dentures and upper and lower full dentures, depending on the type of denture and area of wear.

### Swallowing disorder assessment

To assess subjective dysphagia, 25 items of the Dysphagia Handicap Index (DHI) [12] and 10 items of the Korean version of the Eating Assessment Tool-10 (K-EAT-10) were used [13].

The DHI is a self-report questionnaire that measures difficulty caused by dysphagia. There are 25 items in total: nine items in the physical domain, nine items in the functional domain, and seven items in the emotional domain. Each survey item is rated on a 3-point scale (0 = never, 2 = sometimes, and 4 = always). The DHI score ranges from 0 to 100, with higher raw scores indicating more severe dysphagia. In addition, the K-EAT-10, a clinical assessment scale for dysphagia [13], was used to estimate the patients’ perceived symptoms of dysphagia. The K-EAT-10 is a 10-item self-report questionnaire, with each item describing a problematic aspect of swallowing for patients with dysphagia on a 5-point scale (0 = not a problem at all, from 1 = not a problem to 4 = severe problem) based on the severity of dysphagia. The total K-EAT-10 score ranges from 0 to 40 points, and a higher total score indicates a more severe swallowing disorder. Additionally, swallowing is considered abnormal when the total score is 3 or more.

### Assessment of performance in relation to activities of daily living

The Korean version of the Modified Barthel Index (K-MBI) [14] was used to evaluate the participants’ performance in activities of daily living. The 10 items included were personal hygiene, bathing, feeding, toilet use, stair climbing, dressing, bowel control (defecation), bladder control (voiding), ambulation, chair/bed transfer. The performance on these activities were self-reported on a 5-point scale (1 = not able to perform at all, 2 = needs maximum assistance, 3 = needs moderate assistance, 4 = needs minimal assistance or supervision, and 5 = completely independent) based on the level of assistance required. The Korean version of the Barthel Index is an index that ranges from 0 to 100 points, and a higher score means that an independent daily life is possible.

### Oral hypofunction assessment

To evaluate and determine the decline in oral function and frailty in Korean older adults, 6 items (masticatory ability, occlusal force, tongue strength, salivary gland function, oral cleanliness, and swallowing function) were assessed according to the oral frailty diagnostic criteria announced by the KAGD in 2022. In this study, we evaluated the five items of the KAGD excluding Modified Water Swallowing Test (MWST). Instead of the MWST, we collected through the aforementioned questionnaire (DHI and K-EAT-10) to determine the degree of swallowing difficulty. The evaluation criteria for above 5 items used in the measurement are described in Table 1. The medical device manufacturer’s criteria for determining occlusal pressure, tongue pressure, and oral dryness were used. Oral function tests were performed by three investigators. The investigators were trained, confirming an interrater reliability of 0.995 (*P* < 0.001), to reduce measurement errors among investigators. Oral frailty was determined if functional decline was found in two or more survey items based on KAGD standards.

**Table 1:** Oral hypofunction assessment items and criteria

Item	Assessment method	Criteria
Chewing ability	• Color-changing chewing gum (Masticatory Performance Evaluating Gum XYLITOL, Lotte, Tokyo, Japan) was chewed for 1 min, and the change in color was determined by comparing it to a color chart (range: 1–5).	• Risk: 1, 2 • Normal: 3–5
Maximum occlusal pressure	• The sample was collected by biting a pressure-sensitive film (Dental Prescale II, GC Co., Ltd, Tokyo, Japan) with the maximum intercuspal position for 3 s. • The collected films were analyzed using a a dedicated scanner (GT-X830, EPSON, Tokyo, Japan) and software (Bite Force Analyzer, GC Co., Ltd, Tokyo, Japan).	• Risk: < 500 N • Normal: 500 N or higher
Number of remaining teeth	• Oral examination using a tongue depressor and penlight	• Risk: < 20 • Normal: 20 or more
Maximum tongue pressure	• Participants were asked to bite a JMS tongue pressure measuring instrument (TPM-02, JMS Co., Ltd, Tokyo, Japan) lightly and use maximum force to bite the balloon between the tongue and roof of the mouth for at least 7 s. • A total of three trials were performed to determine the maximum value.	Criteria for 60–69 years old • Risk: ≤ 30 kPa • Normal: greater than 30 kPa Criteria for Over 70 years old • Risk: ≤ 20 kPa • Normal: greater than 20 kPa
Oral dryness	• With the participants’ tongues out, an oral moisture checking device (Mucus, Life Co., Saitama, Japan) was placed perpendicular to the mucosa to measure it with a force of 200 g for 2 s. • The average value of three consecutive measurements was used.	• Risk: < 27.0 • Normal: 27.0 or higher
Oral cleanliness	• Score for the oral cleanliness item on the Oral Health Assessment Tool (0: Clean and no food particles or tartar in the mouth or dentures; 1: Food particles, tartar, or plaque in one to two areas of the mouth or small area of dentures or halitosis (bad breath); 2: Food particles, tartar, or plaque in most areas of the mouth or dentures or severe halitosis (bad breath)	• Risk: 2 • Caution: 1 • Normal: 0

### Statistical analysis

A frequency analysis was performed for the general characteristics of the study participants (age, gender, presence of systemic diseases, BMI, and denture use). Before analyzing differences in oral function status by age, we calculated Pearson’s correlation coefficient among study participants’ age, gender, presence of systemic diseases, BMI, K-MBI, and oral frailty, which may affect research results. To understand the differences in oral function status by age, the ages were grouped into 60 s, 70 s, and 80 s for analysis, and the normality of the collected data was ensured by the Shapiro–Wilk test. One-way analysis of variance was used to determine the level of subjective dysphagia (DHI, K-EAT-10), K-MBI and oral hypofunction (chewing ability, maximum occlusal pressure, number of remaining teeth, maximum tongue pressure, oral dryness, and oral cleanliness) according to age group, and Scheffe’s test was performed for post hoc analysis. The IBM SPSS 21.0 program (SPSS Inc., Chicago, IL, USA) was used to analyze the collected data, and the level of statistical significance was set at 0.05.

## Results

### General characteristics of the participants

Among the 206 people who participated in the study, 105 (72.8%) were women and 93 (45.1%) were seniors in their 80 s, forming the majority of the participants. The number of males and females, categorized by age group, are as follows: in their 60 s, 9 (25.9%) males and 26 (74.3%) females; in their 70 s, 25 (32.1%) males and 53 (67.9%) females; and in their 80 s, 22 (23.7%) males and 71 (76.3%) females. Most participants experienced systemic diseases; approximately 82.5% had at least one systemic disease that they had experienced or were currently experiencing (Table 2). Among the older adults in their 60 s who wore dentures in the upper or lower jaw, four (11.4%) wore partial dentures in the upper jaw and six (17.1%) in the lower jaw, and two (5.7%) wore full dentures in the upper jaw and two (5.7%) in the lower jaw. Among those in their 70 s, nine (11.5%) wore partial dentures in the upper jaw and 11 (14.1%) in the lower jaw, and 14 (17.9%) wore full dentures in the upper jaw and seven (9.0%) in the lower jaw. Finally, among those in their 80 s, 15 (16.1%) wore partial dentures in the upper jaw and 25 (26.9%) in the lower jaw, and 37 (39.8%) wore full dentures in the upper jaw and 17 (18.3%) in the lower jaw (Table 2). After calculating a Pearson’s correlation coefficient, there was no relationship between oral frailty and the participant’s gender, number of systemic diseases, BMI, and K-MBI (Table 3).

**Table 2:** General characteristics of the participants (gender, age, and number of systemic diseases)

	N	%
Gender		
Male	56	27.2
Female	150	72.8
Age		
60 s	35	17.0
70 s	78	37.9
80 s	93	45.1
BMI		
Underweight	5	2.4
Normal	56	27.2
Pre-obesity	48	23.3
Obesity	97	47.1
Number of systemic diseases		
No	35	17.5
One	53	25.7
Two or more	117	56.8
Denture use		
Partial (upper)	28	13.6
Partial (lower)	42	20.4
Full (upper)	53	25.7
Full (lower)	26	12.6

*BMI* body mass index

**Table 3:** Correlation between oral frailty and demographics

Oral frailty	Age	Gender	Systemic diseases	BMI	K-MBI
*r*	0.154^*^	0.000	0.011	-0.090	0.000
*p*-value	0.027	0.998	0.881	0.201	0.995

*r* is the Pearson correlation coefficient

^*^p < 0.05

### Subjective level of dysphagia and ability to perform activities of daily living tasks by age

Based on the analysis of the level of dysphagia by age, participants in their 80 s had a statistically significant higher DHI score than those in their 70 s, indicating more difficulty in swallowing (Table 4). Although there was no significant difference in K-EAT-10 data among the three groups, participants in their 80 s had more difficulty swallowing than the other age groups. Among the DHI sub-scales, the physical domain had the highest scores across all age groups, and the K-EAT-10 scores were all in the normal range with total scores below 3 (threshold for abnormality: 3 or higher).

**Table 4:** Comparison of the levels of dysphagia and independence of daily living by age

	Age				*p*-value
	60 s (*n* = 35)	70 s (*n* = 78)	80 s (*n* = 93)	Total (*n* = 206)	
DHI	8.00 ± 12.64^a,b^	6.38 ± 7.59^a^	11.78 ± 11.68^b^	9.10 ± 10.75	0.003
Physical	3.60 ± 4.08	3.13 ± 3.88	4.62 ± 4.81	3.88 ± 4.39	
Functional	3.31 ± 4.80	2.38 ± 3.94	3.61 ± 4.36	3.10 ± 4.30	
Emotional	2.51 ± 4.98	1.54 ± 2.75	2.49 ± 3.51	2.14 ± 3.56	
K-EAT-10	2.06 ± 5.77	1.08 ± 2.12	2.22 ± 4.08	1.76 ± 3.87	0.140
K-MBI	99.29 ± 2.27	99.41 ± 1.81	98.57 ± 3.04	99.01 ± 2.53	0.074

Data obtained from one-way analysis of variance test and Scheffe’s post hoc test between groups

*DHI* Dysphagia Handicap Index, *K-EAT-10* Korean version of Eating Assessment Tool-10, *K-MBI* Korean Version of the Modified Barthel Index

^a,b^Different superscript letters indicate significant differences between groups

The participants’ ability to perform activities of daily living tasks, with the scores averaging at 99.7 and a high proportion of participants in all age groups reporting complete independence in their activities of daily living (K-MBI) (Table 4). There was no correlation between the K-MBI and oral frailty among the participants in this study (Table 3).

### Comparison of the levels of oral hypofunction by age

Oral function in terms of chewing ability (maximum occlusal pressure, number of remaining teeth), maximum occlusal pressure, and maximum tongue pressure (Table 5, *p* < 0.05) declined with increasing age. Chewing ability assessments showed a slight decrease in chewing ability with increasing age but were within the normal range for all ages, whereas occlusal and tongue pressures were at risk of oral frailty in all age groups, indicating that oral hypofunction decreased with age.

**Table 5:** Comparison of the levels of oral hypofunction by age

	Age				*p*-value
	60 s (*n* = 35)	70 s (*n* = 78)	80 s (*n* = 93)	Total (*n* = 206)	
Chewing ability	4.34 ± 0.80^a^	4.06 ± 1.21^a^	3.34 ± 1.26^b^	3.79 ± 1.24	0.000
Maximum occlusal pressure	426.93 ± 294.47^a^	406.21 ± 311.91^a^	256.94 ± 252.63^b^	342.34 ± 292.66	0.001
Number of remaining teeth	23.51 ± 6.73^a^	21.06 ± 9.41^a^	13.47 ± 11.04^b^	18.05 ± 10.66	0.000
Maximum tongue pressure	29.8 ± 7.68^a^	27.39 ± 7.84^a^	18.93 ± 8.65^b^	23.98 ± 9.39	0.000
Oral dryness	27.54 ± 2.02	27.23 ± 2.12	26.72 ± 2.38	27.05 ± 2.24	0.120
Oral cleanliness	0.77 ± 0.65	0.82 ± 0.58	0.98 ± 0.64	0.88 ± 0.62	0.128

Data obtained from one-way analysis of variance test and Scheffe’s post hoc test between groups

^a,b^Different superscript letters indicate significant differences between groups

On the other hand, the results of the oral mucosal wetness and oral hygiene maintenance tests, which tested variables related to salivary gland function (oral dryness), did not differ significantly by age. However, according to the judgment criteria, participants in their 80 s had a dry mouth, and their oral cleanliness was close to the degree of oral frailty requiring caution.

## Discussion

As the proportion of the older adult population increases with time, efforts are needed to understand the process of frailty and systemic changes that may occur in older adults to ensure their quality of life [15]. Frailty in the older adult population requires attention because of the decreased physiological reserve to maintain homeostasis due to a general decline in physical functioning, resulting in an inability to respond appropriately to external stresses, which can have negative consequences in terms of increased morbidity for various diseases, disability, dependence, falls, long-term care, and mortality [16, 17]. Thus, there is also a growing interest in oral health, as the frequency of systemic frailty is 2.06 times higher in older adults with oral hypofunction [18]. Therefore, identifying the level of oral hypofunction with age and suggesting appropriate management interventions will be essential to enable older adults to lead healthy lives.

In this study, 217 older adults living in Wonju, Gangwon State, South Korea, who visited a senior citizen community center were assessed for dysphagia and independence in daily life, evaluating their oral hypofunction. The older adults in Korea are increasing rapidly, particularly in Gangwon State, where over 47.2% of the residents are 65 years or older. This has resulted in a significant aging issue, and Gangwon State has already become a super-aged society in 2020. To address these geographical characteristics, we conducted a study in Wonju, Gangwon State. We gave priority to the regions with a high percentage of elderly residents and contact to senior centers in those areas to get permission for their participation in the study. Therefore, they seemed to be a suitable sample for the presentation of the appropriate management methods that reflect the proportion of older adults (aged ≥ 65 years) in the Korean population and physical changes and characteristics of older adults in the future.

Across all age groups, the average DHI score was 9.1 out of 100, and the average K-EAT-10 score was an average of 1.7 out of 40, indicating that the participants’ subjective level of dysphagia was not high. A total score of 3 or higher on the K-EAT-10 was considered to indicate oral hypofunction [5], and even the oldest group, with an average score of 2.22, was found to be at a healthy level. This may have been due to the fact that the participants in this study had a level of health that allowed them to visit the senior citizen community center. The K-MBI value of the participants in this study averaged at 99.7 out of 100, indicating that they were independent and generally healthy, which may have influenced their subjective assessment. In addition, based on the trend of health insurance in dental care in Korea over the past decade, as health insurance coverage has expanded, the number of older adults with dentures or implant prosthetics has been increasing, and their masticatory function has been restored; thus, subjective swallowing difficulties and discomfort may not have been as pronounced [19, 20]. Nevertheless, according to the analysis of dysphagia assessment indicators by age, those in their 80 s were more likely to experience difficulty swallowing during daily activities based on both the DHI and K-EAT-10 scores compared with those in their 60 s and 70 s (Table 4). Older adults in their 80 s scored particularly high on the physical domain of the DHI. The physical domain included coughing when consuming food and complaining of difficulty swallowing due to dry mouth. The higher scores in this domain of older adults in their 80 s may reflect the findings that chewing ability, maximum occlusal pressure, number of remaining teeth, and maximum tongue pressure decreased with age (Table 5, *p* < 0.05). In particular, in the occlusal pressure assessment, older adults in their 80 s had significantly reduced maximum occlusal pressure, averaging at 256.9 N, compared with those in their 60 s (426.9 N) and 70 s (406.2 N). For older adults in their 80 s, the number of remaining teeth also averaged at 13.5, approximately 6.5 fewer than 20, which would be considered to be at a risk level in an oral frailty assessment. Chewing ability is affected by the number of remaining natural teeth. One study has reported decreased chewing ability with decreased number of remaining teeth in older adults in their 80 s, leading to poor nutrition, health, and quality of life [21]. Therefore, encouraging the older adult population to visit the dentist for regular check-ups to detect and treat dental caries and periodontal disease at an early stage, which are the main causes of tooth loss, can have a positive impact on not only preventing oral diseases in the older adult population but also improving systemic health and quality of life.

As for tongue pressure, the Japanese Society of Gerodontology reported in 2016 [5] that a maximum tongue pressure of less than 30 kPa was considered a risk regardless of age. However, in this study, a maximum tongue pressure of less than 30 kPa was assessed as a risk factor for those in their 60 s and a maximum tongue pressure of less than 20 kPa for those in their 70 s and 80 s, according to the manufacturer’s instructions for the tongue pressure tester. The results of this study showed that tongue pressure tended to decrease with age, with an average of 18.9 kPa in older adults in their 80 s. As older adults lose muscle throughout the body in response to aging, the tongue, masticatory, and pharyngeal muscles are also known to lose muscle mass and function, eventually affecting swallowing [22–24]. This may explain why reduced tongue strength was observed in older adults in their 80s. As the decline in tongue strength is expected to be a consequence of the loss of muscle mass during the aging process, strategies to improve tongue strength are needed to improve dysphagia in older adults. Previous studies have reported that stimulating the tongue, including through tongue-to-palate resistance training (TPRT), can improve muscle strength and is recommended as a strategy to improve swallowing in patients with dysphagia [25, 26]. In addition to TPRT, various devices have been developed to improve tongue strength and are available for purchase; however, formalized guidelines for oral function improvement considering the characteristics of older adults are still lacking. Oral function in older adults is affected by age, gender, and systemic diseases [27]; however, criteria for evaluating oral hypofunction have not been proposed. In particular, an oral hypofunction assessment considering the age and gender of Korean older adults have not been performed. Therefore, it is necessary to consider age and gender rather than uniform criteria when developing Korean-style oral dysfunction evaluation criteria; thus, the results of this study can be used as an important basis for proposing evaluation criteria [28].

On the other hand, in the oral mucosal wetness test for determining salivary gland function (oral dryness), a significant difference between the age groups was not observed; however, participants in their 60 s and 70 s had oral mucosal wetness within the normal range (27.0 or higher), whereas those in their 80 s had an oral mucosal wetness of 26.7, indicating a decrease in salivary gland function. Overall, the salivary gland function of older adults in this study tended to be lower compared to the mean of 29.7 ± 2.0 reported in a study that measured salivary gland function in older adults aged 65 years and older (mean age: 73.6 ± 8.2) who visited a dentist [29]. Decreased salivary gland function is one of the major oral symptoms found in older adults, and age has been reported to be one of the largest factors contributing to differences in oral fluid volume [30]. Furthermore, 82.5% of the older adult participants in this study were experiencing systemic diseases, and many studies have reported that medications for the management of systemic diseases experienced by older adults have an impact on salivary gland function [31–33]. Especially considering that the incidence of systemic diseases increased with age, the proportion of participants with dry mouth was higher among older adults in their 80 s than in those in their 60 s and 70 s.

In Korea, in 2022, the Korean Academy of Geriatric Dentistry announced the criteria for diagnosing oral frailty based on a total of six items, with an oral hypofunction observed in two or more of the five items examined in this study (poor oral hygiene, oral dryness, decreased occlusal pressure, decreased tongue pressure, and decreased chewing ability), as well as the MWST, after evaluating the oral function status of older adults [11]. In our study, the total number of older adults who could be judged as having oral frailty when oral hypofunction was observed in two or more items, for the five items except MWST, was 92 (44.7%). By age, 31.4% of those in their 60 s, 43.6% of those in their 70 s, and 50.5% of those in their 80 s were evaluated to have oral frailty, indicating that the proportion of older adults with oral frailty increased with increasing age. In this study, information on MWST was obtained from the DHI and K-EAT-10 and was found to be good overall; hence, it was not included in the determination of oral frailty. In addition, in the course of this study, the MWST was evaluated in some older adults (*N* = 100), and the average score was 4.9 ± 0.4 out of a total of 5 (the standard for normal was 4 or higher), indicating that most of them were at a healthy level (data not shown); hence, it did not seem to have a significant effect on judging oral frailty in Koreans. The Japanese Society of Gerodontology evaluated the above six items and suggested diagnosing oral frailty if 3 or more items were at the “risk” level, and it was reported that about 22.5% of the elderly in Japan experienced oral frailty [34].

Despite the difficulties in making direct comparisons, a large number of older adults participating in this study, most of them systemically healthy, seemed to experience oral frailty. Among previous studies, Cruz-Moreira et al. [18] found that oral frailty was more prevalent in women than in men, and Kugimiya et al. [34] reported an 11.4% prevalence of oral frailty in older adults in their 60 s, compared with 43.9% in 85-year-olds, suggesting that the prevalence of oral frailty increased with age. In other words, the fact that the participants in this study were mostly women (72.8%) and the percentage of older adults in their 80 s was high (45.1%) may have contributed to the high rate of oral frailty among the participants in this study.

Currently, there is no systematic intervention plan to improve the oral frailty or oral function. However, a study conducted on older adults who had poor oral function showed significant improvement in their oral function after undergoing a regimen of oral exercises, mouth-opening training, tongue-pressure training, articulation training, and masticatory training [35]. Based on these findings, this study intended to suggest aspects should be considered when developing interventions to improve oral function in Korean older adults. As a result of comparing the oral frailty diagnostic items, the number of participants with oral hypofunction was higher compared to those reported with hypofunction on other assessment items, with 154 participants (74.8%) having a decline in occlusal pressure and 147 (71.4%) having a decline in tongue pressure. In particular, 79.6% of the participants in their 80 s had a decrease in occlusal pressure, and 60.2% had fewer than 20 residual natural teeth, indicating that the proportion of participants in their 80 s experiencing a decrease in chewing ability was greater than 5% and that 86.7% were at risk in terms of tongue pressure (data not shown). Therefore, the first items to be considered for improving oral function are 1) early detection of dental and periodontal diseases to improve chewing ability and regular dental visits to prevent accumulation of diseases with age, 2) appropriate rehabilitation exercises to improve tongue pressure to prevent and alleviate dysphagia, and 3) presenting customized diets, which may reduce the overall proportion of older adults with oral hypofunction in the future.

There are several limitations in this study. First, this study was conducted with older adults in their 60 s and older living in Wonju, and most of the participants were healthy with no difficulties in daily living or mobility. Thus, the rate of oral frailty or deterioration reported here may be lower than that of hospitalized patients or residents of long-term care facilities. Therefore, the generalizability of the study findings to all older adults in Korea is limited. Secondly, although various criteria have been proposed to evaluate oral frailty [5, 36–38], this study used the criteria proposed by the KAGD. Therefore, the distribution of oral frailty by age may vary depending on the evaluation criteria. The Korean oral frailty diagnosis and clinical practice guidelines proposed by KAGD were developed for the purpose of application in primary medical settings such as dental clinics and hospitals for the older adults, and studies published in various countries were referred to during the development process. In this study, oral frailty was evaluated by comprehensively considering the evaluation standards proposed by the KAGD and the instructions provided by the manufacturer. Thus, it is clinically valid to differentiate oral function differences according to age in older adults. Lastly, this study did not provide detailed information on the demographic characteristics and general health status of the subjects. However, it should be considered that the older adults who participated in the study were able to move around and live in similar residential areas, which minimizes the impact of these characteristics on the study results. Our research team focused on determining the oral function of the older adults and developing expert intervention methods for healthy aging. Thus, it is important to consider demographic characteristics and systemic conditions more comprehensively for better development in further study. Nevertheless, the significance of the findings of this study is that by objectively identifying changes in oral function with aging, the current status of oral hypofunction with respect to age in Korean older adults was identified, providing evidence in support of the need for oral rehabilitation. Many countries have advocated the need for oral rehabilitation training to address oral hypofunction. In Japan, upper limb joint mobility training, head joint mobility training, breathing training, salivary gland massage, lip muscle training, and tongue muscle training are provided to improve oral function [4, 35]. Proper training has been found to be effective in improving multiple components of oral function. In Korea, oral hypofunction is partially addressed in dysphagia rehabilitation by occupational therapists. In dentistry, the concept of oral frailty has recently been introduced, with various clinical specialists making efforts to prevent oral deterioration in older adults by operating oral function rehabilitation programs [11, 39, 40]. However, appropriate rehabilitation that considers the level of oral hypofunction and age of older adults is still lacking. Therefore, a multidisciplinary approach will be needed to identify and share gaps in evidence-based systematic interventions for the early detection and management of oral frailty symptoms, which is expected to contribute to the improvement of oral function and overall quality of life in older adults.

## Conclusions

The findings of this study suggest that increasing age is associated with dysphagia and oral hypofunction in older adults. To manage oral health early on and improve oral health, it is important to monitor changes in the oral function such as deterioration in oral health status, chewing and swallowing disorders, deterioration of oral motor skills, and oral pain. The study’s results can help develop individualized intervention plans, including standardizing oral hypofunction items according to older adults’ age and providing dental treatment, myofunctional rehabilitation programs, and customized diets.

## Data Availability

The datasets used/analyzed during the current study are available from the corresponding author upon reasonable request.

## Abbreviations

BMI: Body Mass Index
DHI: Dysphagia Handicap Index
K-EAT-10: Korean version of Eating Assessment Tool
KAGD: Korean Academy of Geriatric Dentistry
K-MBI: Korean version of the Modified Barthel Index
MWST: Modified Water Swallowing Test
TPRT: Tongue-to-palate resistance training
